# Supplementary material for: The causal role of circulating inflammatory markers in osteoporosis: a bidirectional Mendelian randomized study
Source: Front Immunol. 2024 Jul 18;15:1412298. doi: 10.3389/fimmu.2024.1412298 (PMC11291241; doi:10.3389/fimmu.2024.1412298)
Supplement: Supplementary file 1 [file DataSheet_1.zip › supplementary material/OSTEOPOROSIS-CIM/GCST90274824.tsv.gz_forest.pdf]

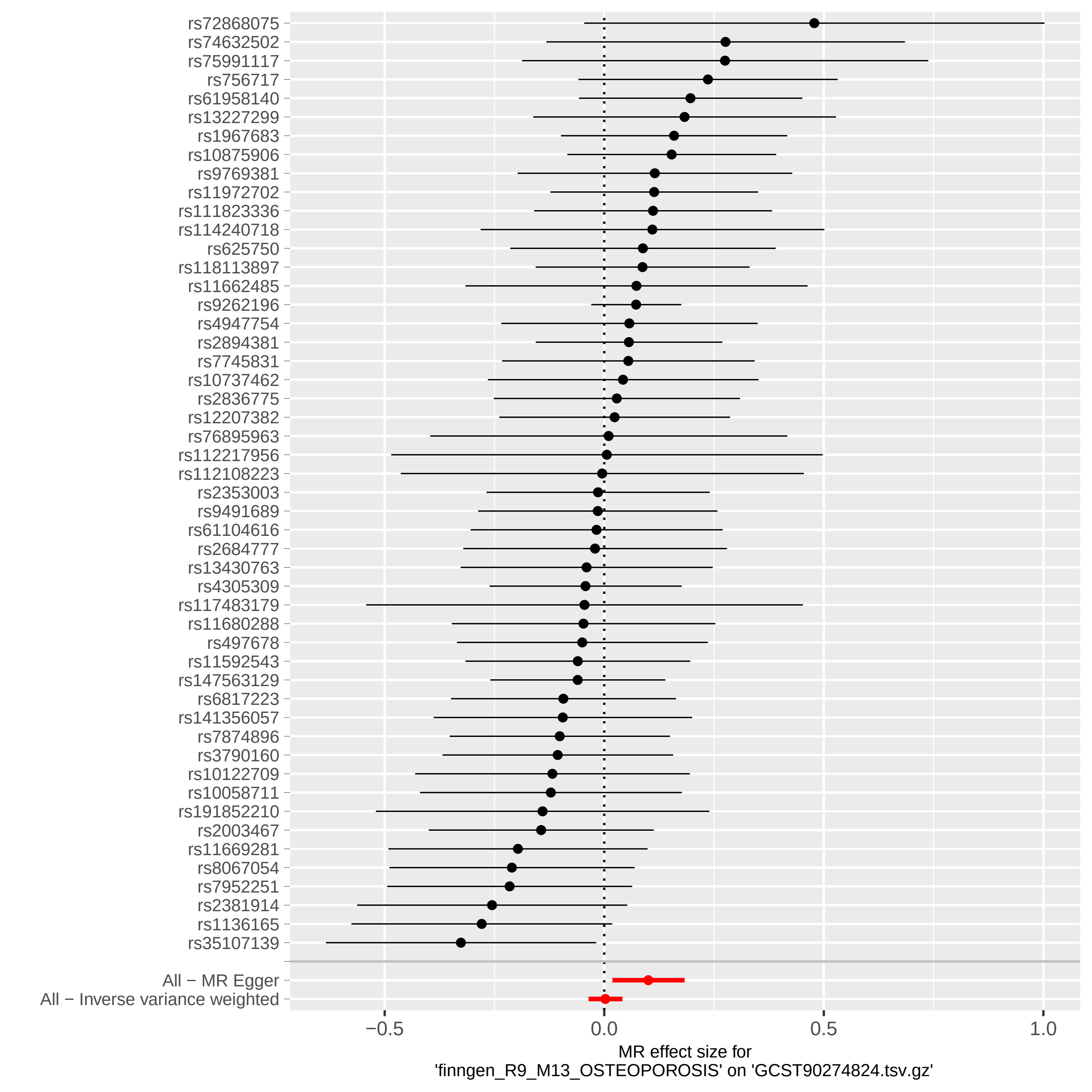
rs72868075
rs74632502
rs75991117
rs756717
rs61958140
rs13227299
rs1967683
rs10875906
rs9769381
rs11972702
rs111823336
rs114240718
rs625750
rs118113897
rs11662485
rs9262196
rs4947754
rs2894381
rs7745831
rs10737462
rs2836775
rs12207382
rs76895963
rs112217956
rs112108223
rs2353003
rs9491689
rs61104616
rs2684777
rs13430763
rs4305309
rs117483179
rs11680288
rs497678
rs11592543
rs147563129
rs6817223
rs141356057
rs7874896
rs3790160
rs10122709
rs10058711
rs191852210
rs2003467
rs11669281
rs8067054
rs7952251
rs2381914
rs1136165
rs35107139
All − MR Egger
All − Inverse variance weighted
−0.5
0.0
0.5
1.0
MR effect size for
'finngen_R9_M13_OSTEOPOROSIS' on 'GCST90274824.tsv.gz'
